# Understanding addiction in e-cigarette users – the EVAPE project

**DOI:** 10.1093/eurpub/ckac130.078

**Published:** 2022-10-25

**Authors:** V Lohner, S Schneider, M Andreas, D Szafran, N Grundinger, S Vollstädt-Klein, GT Fong, A McNeill, U Mons

**Affiliations:** 1 Department of Cardiology, University of Cologne, Cologne, Germany; 2 Medical Faculty Mannheim, University of Heidelberg, Mannheim, Germany; 3 Department of Psychology, University of Waterloo, Waterloo, Canada; 4 School of Public Health Sciences, University of Waterloo, Waterloo, Canada; 5 Ontario Institute for Cancer Research, Toronto, Canada; 6 Psychology and Neuroscience, King’s College London, London, UK; 7 SPECTRUM, London, UK

## Abstract

**Background:**

Electronic cigarettes (e-cigarettes) are often advertised as a healthier option to combustible cigarettes and as smoking cessation aid. However, e-cigarettes are a growing health concern and their addictive potential remains to be fully understood. Within the EValuation of the Addictive Potential of E-cigarettes (EVAPE) project, we studied subjective and objective measures of addiction in relation to e-cigarette use.

**Methods:**

This cross-sectional analysis was based on 832 participants of the first wave (2016) of England from the ITC Four Country Smoking and Vaping (4CV) Survey, who were using e-cigarettes daily or weekly for at least four months. Perceived addiction to e-cigarettes was categorised as very vs. not/somewhat addicted, and perceived addictiveness of e-cigarettes relative to combustible cigarettes as equally/more addictive vs. less addictive. Objective measures of addiction included urge to vape, time to first vape after waking, frequency of use, and used nicotine strength. We examined associations between these objective and subjective measures of addiction using multivariate logistic regression, adjusted for age, gender, education, and cigarette smoking.

**Results:**

17.8% of participants reported feeling very addicted to e-cigarettes and 42.3% considered e-cigarettes equally/more addictive than combustible cigarettes. Those who felt very addicted had higher odds of regarding e-cigarettes as more addictive (OR 3.43 (95%-CI 2.29-5.19)). All objective measures of addiction were associated with higher perceived addiction, whereas only a shorter time to first vape was associated with perceived product addictiveness.

**Conclusions:**

Subjective measures of addiction to e-cigarettes, in particular perceived addiction, correspond with objective measures. Understanding the addictive potential of e-cigarettes is the cornerstone for developing new strategies for prevention and treatment, and ultimately understanding their role from a public health perspective.

**Key messages:**

- Despite being promoted as healthier alternative to combustible cigarettes, electronic cigarettes are a growing health concern, also given their potential addictiveness.
- We found both subjective and objective indicators of addiction to be prevalent in a relevant proportion of electronic cigarette users, suggesting an addictive potential of electronic cigarettes.

